# Preparation and surface functionalization of MWCNTs: study of the composite materials produced by the interaction with an iron phthalocyanine complex

**DOI:** 10.1186/1556-276X-6-353

**Published:** 2011-04-20

**Authors:** Esther Asedegbega-Nieto, María Pérez-Cadenas, Jonathan Carter, James A Anderson, Antonio Guerrero-Ruiz

**Affiliations:** 1 Departamento de Química Inorgánica y Técnica, Facultad de Ciencias, UNED, Paseo Senda del Rey no. 9, 28040 Madrid, Spain; 2 Surface Chemistry and Catalysis Group, Department of Chemistry, University of Aberdeen, Regent Walk, Aberdeen, UK

## Abstract

Carbon nanotubes [CNTs] were synthesized by the catalytic vapor decomposition method. Thereafter, they were functionalized in order to incorporate the oxygen groups (OCNT) and subsequently the amine groups (ACNT). All three CNTs (the as-synthesized and functionalized) underwent reaction with an iron organometallic complex (FePcS), iron(III) phthalocyanine-4,4",4",4""-tetrasulfonic acid, in order to study the nature of the interaction between this complex and the CNTs and the potential formation of nanocomposite materials. Transmission electronic microscopy, N_2 _adsorption at 77 K, thermogravimetric analysis, temperature-programmed desorption, and X-ray photoelectron spectroscopy were the characterization techniques employed to confirm the successful functionalization of CNTs as well as the type of interaction existing with the FePcS. All results obtained led to the same conclusion: There were no specific chemical interactions between CNTs and the fixed FePcS.

## Introduction

Metallophthalocyanines possess unique physicochemical, electronic, and electrocatalytic properties, making them useful in various application fields. There is vast literature regarding their use as sensors [1-3] as their properties are readily modified by the presence of certain molecules. The possibility of depositing these phthalocyanine complexes as thin films compatible with microelectronic devices is another driving force for this purpose. Another use is as electrocatalysts in the reduction of oxygen as they can overcome the spin barrier and provide a low-energy route for the highly stable dioxygen to react, thanks to the redox potential of the metal in the phthalocyanine [4]. These complexes have also been employed as oxidation catalysts owing to (1) the resemblance of their macrocyclic structure with that of porphyrins widely used by nature in the active sites of oxygenase enzymes; (2) their rather cheap and facile preparation on a large scale; and (3) their chemical and thermal stability [5].

There are various studies involving the fixation of phthalocyanine complexes onto different supports. The composites of metal phthalocyanines/carbon nanotubes [CNTs] have inspired considerable research interest because of their high quantum efficiency facilitated by the charge transfer between them and the complementary properties of the composites. The resulting metallophthalocyanine/CNT complexes possess the unique properties of phthalocyanine without any destruction of electronic properties and structures of CNTs.

An important aspect to be considered is the interaction between the metallophthalocyanine complex and the CNT. Several authors claim covalent bonding for certain metallophthalocyanines, while non-substituted complexes would be non-covalently adsorbed onto the carbon nanotubes *via π*-*π *interactions [6,7]. In this work, we study the introduction of different surface groups onto CNT and their effect on the interaction between the carbon material and an ionic iron phthalocyanine.

### Experimental procedure

CNTs were synthesized by the catalytic vapor decomposition method. The reaction setup and conditions are described elsewhere [8]. The CNTs obtained were chemically treated in order to functionalize the surface. This consisted of a two-step procedure. Firstly, the originally prepared CNTs were oxidized with HNO_3 _(65 wt.%, 363 K, 72 h), thereby obtaining oxidized CNT, which was further treated with an amine (ethylenediamine in *n*-hexane, 343 K, 24 h) to give the aminated CNT [ACNT]. The as-synthesized and treated CNTs were reacted with a commercially available iron(III), phthalocyanine-4,4",4",4""-tetrasulfonic acid (FePcS), which is a hydrated monosodium salt compound that contains oxygen (Sigma-Aldrich, St. Louis, MO, USA), in order to obtain three composites, FePcS/CNTs (of 5 wt.% Fe). The procedure involved stirring 200 mg of carbon nanotubes in an aqueous solution of FePcS for 17 h at room temperature. After that, the solvent was evaporated and the solid dried, 373 K for 18 h.

Various analyses were carried out in order to fully characterize the prepared CNTs as well as the corresponding composites. Transmission electronic microscopy [TEM] was performed on synthesized CNTs employing a JOEL JEM 2000FX system. Surface area and pore size distribution were determined from N_2 _adsorption at 77 K (Micromeritics ASAP 2000 surface analyzer). Samples were previously degassed at 393 K for 5 h. Thermogravimetric analysis data were collected using a SDTQ600 5200 TA system. The samples were heated under an inert helium and air atmosphere (1,273 K, 10 K min^-1^). Temperature-programmed desorption [TPD] experiments were performed under vacuum in a quartz reactor coupled with a mass spectrometer (Baltzers, QMG 421, 1,100 K, 10 K min^-1^). The surface of the CNTs and composites was analyzed by X-ray photoelectron spectroscopy [XPS] with an Omicron spectrometer system equipped with a hemispherical electron analyzer operating in a constant pass energy using Mg Kα radiation (hν = 1,253.6 eV). C 1 s, O 1 s, N 1 s, Na 1 s, and Fe 2p_3/2 _individual high-resolution spectra were measured. All binding energies were referenced to C 1 s line at 284.6 eV.

## Results and discussion

### Carbon nanotubes

**Figure 1:**
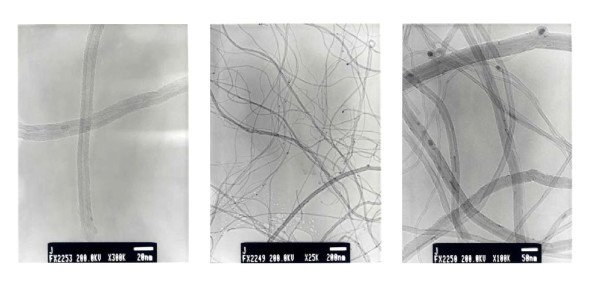
**TEM images of originally synthesized CNTs**. TEM, transmission electron microscopy.

TEM results can be viewed in the micrographs of Figure 1. As can be seen, the obtained carbon material consists of bundles of multiwalled CNTs of varying diameters (generally between 10 and 20 nm).

Nitrogen adsorption isotherms at 77 K showed that all CNTs displayed type II isotherms, which implies that the samples contain mesopores and macropores [9]. Brunauer-Emmett-Teller [BET] surface areas and mesopore volumes are summarized in Table 1. As can be seen, CNT has a surface area of 90 m^2^/g, which increased to 120 m^2^/g after surface oxidation. This is most likely due to the elimination of some retained Fe particles at tube ends by the HNO_3 _solution, thereby facilitating the N_2 _access to the interior of the tubes. This opening, confirmed by TEM (not presented here for the sake of brevity), would be responsible for the increase in surface area. However, after functionalizing these oxidized CNT surfaces with ethylenediamine, the surface area was lowered to 82 m^2^/g, possibly due to restricted N_2 _access resulting from the coverage by the amine.

**Table 1:** Characterization properties of CNTs

Sample	BET morphological parameters		TGA: residual weight (%)		XPS at.% O 1 s
	***S***_**BET **_**(m**^**2**^**/g)**	***V***_**mesopores **_**(cm**^**3**^**/g)**^**a**^	CNTs	**FePcS/CNTs**^**b**^	
CNT	90	0.08	1.68	20.66	2.36
OCNT	120	0.11	4.88	22.17	7.69
ACNT	82	0.09	7.65	24.30	4.08

^a^Volume of mesopores has been calculated by the difference between the volume of N_2 _adsorbed at *P*/*P*° = 0.9 and *P*/*P*° = 0.2.

^b^Residual weight of FePcS/CNT composites.

From the residual weights of materials after heat treatment under helium, an increase in weight loss (due to the elimination of surface groups) after the oxidation process can be observed, and this increase is greater after amination. This would indicate that modification of the surface of the prepared CNTs was successful.

**Figure 2:**
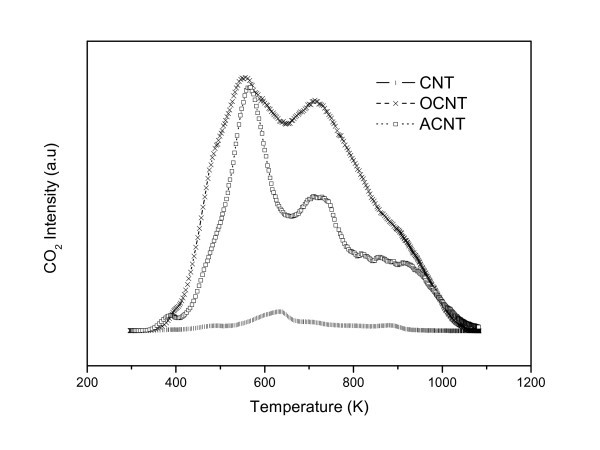
**TPD profiles of the evolution of CO_2_**. TPD, Temperature-programmed desorption.

The evolution of CO and CO_2 _was followed by TPD experiments for all three CNT samples. The profiles obtained gave an estimate of the surface oxygen-containing groups. A significant increase was observed after oxidation treatment of the synthesized CNTs, which reveals that this chemical oxidation is an efficient way to facilitate oxygen incorporation into CNTs. On the other hand, the amination of OCNT gave rise to a reduction in the intensity of CO and CO_2 _peaks due to the decrease in the surface oxygen groups at the expense of the newly formed amine groups. These TPD results suggest that there is indeed interaction between the acidic oxygen groups of the OCNT and the ethylenediamine in the production of ACNT (Figure 2).

This variation noted in oxygen surface groups is further confirmed upon observing the XPS results of O 1 s. In the first place, there was a significant increase in the atomic percent of oxygen (Table 1) after oxidizing the originally prepared CNTs. This oxygen content is strongly reduced after reacting OCNT with the amine due to the formation of the corresponding amide. This provides insight into the type of interaction existing between acidic oxygen groups and basic amine. This could be further elucidated after deconvolution of the O 1 s envelope into three peaks [10]. There was a noticeable decrease (to almost half its initial value) in the COOH peak (at about 534.4 eV) when comparing ACNT with OCNT, which suggests acid/base reaction to form water and the amide function. The nitrogen functional group at ACNT is further confirmed by the presence of a N 1 s peak at 399.9 eV.

### FePcS/CNT composites

The same characterization techniques also gave very valuable information in the study of the prepared composites. Table 1 also collects thermogravimetric [TG] results for the composite where there is a reduction in the weight, which follows the same tendency as in the case of the starting CNTs. Subtracting from this weight percent loss that of the corresponding CNT, an estimate of the weight loss due to the incorporated complex can be made. For all three composites, this value (17-19%) is effectively constant, implying that similar quantities of the Fe complex have been fixed on the carbon substrate. These TG analysis experiments under inert gas conditions also reveal that desorption/decomposition of the retained FePcS complexes take place at the same temperatures in the three studied composites (TG profiles are not shown for the sake of brevity). An obvious conclusion is that the interactions of the FePcS molecules with the CNT surfaces do not depend on the previous functionalization of the carbon nanotube surfaces.

This is also supported by the XPS results where an increase in atomic percent of Fe in the composite with respect to that of the corresponding CNT can be attributed to the presence of the Fe complex in the composite. This difference of about 0.15 is similar in all cases, indicating that the amount of FePc at the surface of each CNT is the same. The anchoring of the Fe complex at the CNT surface was also evidenced by the presence of the S 2p and Na 1 s peaks. On the other hand, binding energy values of the different components of FePcS gave information on changes in the pure complex due to its interaction with the CNT surface. The N 1 s spectrum of phthalocyanines consists of one main peak at 399.0 eV accompanied by a less intensive peak at 400.6 eV. The main peak can be ascribed to the two chemically non-equivalent nitrogens (four central nitrogens and four aza nitrogens), while the other peak is attributed to a shake-up satellite [11]. In FePcS/CNT and FePcS/OCNT composites, similar band shape and position were observed, indicating that N was present in the same chemical environment as in its original pure commercial FePcS. As for FePcS/ACNT, these two peaks were also present, although the proportions changed. The peak at higher binding energy is significantly more intense owing to the participation of new amino functional groups formed on the samples that are overlapped by the shake-up peak. Fe 2p_3/2 _had binding energies of 710.20-710.95 eV in all three samples, and these values are similar to that expected for FePcS [12]. This, together with the doublet separation of about 13.5 eV [13], confirms that Fe remains in its (+III) oxidation state after composite formation. Therefore, FePcS in the composite displays no significant difference with respect to the original pure complex. It seems that interactions between this complex and the CNT are quite weak, do not cause any chemical modification in the complex, and are independent of the surface functionalization of the support.

## Conclusions

The functionalization of CNTs was successful and significant amounts of oxygen surface groups and amine groups were introduced into OCNT and ACNT, respectively. Characterization of the composites gave very valuable information. Firstly, independent of the presence of surface groups, the amount of fixed FePcS is practically the same for all three CNTs. Secondly, the chemical properties of this complex remain unchanged in the composites. These two conclusions are indicative of the interactions between ionic FePcS and surface-modified CNTs. There seems to be no specific chemical interaction, and the weak *π*-*π *interactions are not influenced by the presence of the functional groups on the CNT surface.

## Competing interests

The authors declare that they have no competing interests.

## Authors' contributions

EAN carried out part of the characterization of CNT materials, the interpretation of experimental data as well as writing up of this manuscript. MPC and JC were responsible for synthesis and other characterizations of CNT materials as well as interpretation of experimental data. JAA and AGR participated in the supervision and aided in the result discussion and manuscript revision. All authors read and approved the final manuscript.
